# Syndecan-4 Mediates the Cellular Entry of Adeno-Associated Virus 9

**DOI:** 10.3390/ijms24043141

**Published:** 2023-02-05

**Authors:** Anett Hudák, Matthew Roach, Dávid Pusztai, Aladár Pettkó-Szandtner, Annamária Letoha, László Szilák, Mimoun Azzouz, Tamás Letoha

**Affiliations:** 1 Pharmacoidea Ltd., H-6726 Szeged, Hungary; 2 Department of Neuroscience, Sheffield Institute for Translational Neuroscience (SITraN), University of Sheffield, Sheffield S10 2HQ, UK; 3 Biological Research Centre, Institute of Plant Biology, H-6726 Szeged, Hungary; 4 Albert Szent-Györgyi Clinical Center, Department of Medicine, Faculty of Medicine, University of Szeged, H-6726 Szeged, Hungary; 5 Szilák Laboratories Ltd., H-6725 Szeged, Hungary

**Keywords:** AAV9, cellular entry, gene delivery, heparan sulfate proteoglycans, syndecans

## Abstract

Due to their low pathogenicity, immunogenicity, and long-term gene expression, adeno-associated virus (AAV) vectors emerged as safe and efficient gene delivery tools, over-coming setbacks experienced with other viral gene delivery systems in early gene therapy trials. Among AAVs, AAV9 can translocate through the blood-brain barrier (BBB), making it a promising gene delivery tool for transducing the central nervous system (CNS) via systemic administration. Recent reports on the shortcomings of AAV9-mediated gene delivery into the CNS require reviewing the molecular base of AAV9 cellular biology. A more detailed understanding of AAV9’s cellular entry would eradicate current hurdles and enable more efficient AAV9-based gene therapy approaches. Syndecans, the transmembrane family of heparan-sulfate proteoglycans, facilitate the cellular uptake of various viruses and drug delivery systems. Utilizing human cell lines and syndecan-specific cellular assays, we assessed the involvement of syndecans in AAV9’s cellular entry. The ubiquitously expressed isoform, syndecan-4 proved its superiority in facilitating AAV9 internalization among syndecans. Introducing syndecan-4 into poorly transducible cell lines enabled robust AAV9-dependent gene transduction, while its knockdown reduced AAV9’s cellular entry. Attachment of AAV9 to syndecan-4 is mediated not just by the polyanionic heparan-sulfate chains but also by the cell-binding domain of the extracellular syndecan-4 core protein. Co-immunoprecipitation assays and affinity proteomics also confirmed the role of syndecan-4 in the cellular entry of AAV9. Overall, our findings highlight the universally expressed syndecan-4 as a significant contributor to the cellular internalization of AAV9 and provide a molecular-based, rational explanation for the low gene delivery potential of AAV9 into the CNS.

## 1. Introduction

The advancement of gene therapy is revolutionizing medicine [1,2,3,4,5]. The efficacy of gene therapy approaches relies on the targeted and efficient delivery of functional genes into cells with flawed endogenous genes [6]. Due to their low pathogenicity, immunogenicity and long-term gene expression, adeno-associated virus (AAV) vectors emerged as a safe and efficient tool for gene delivery, hence overcoming safety concerns and setbacks experienced with other viral gene delivery systems in the early gene therapy trials [7,8,9]. Exploring key aspects of AAV biology, including genome configuration and composition, DNA replication and transcription, infectious latency and virion assembly, enabled the efficient cloning and genetic manipulation of the AAV genome, thus making recombinant AAVs (rAAVs) the leading gene delivery platform for clinical gene therapies [10,11,12,13,14,15,16,17,18,19,20,21,22,23,24,25,26,27]. Although rAAV-based gene therapy products have already been approved in the EU and the US, several challenges still exist [10,28,29,30,31].

AAV is a non-pathogenic parvovirus discovered initially as a cell culture contaminant, just like its most frequently utilized serotype, AAV2 [10,32]. The non-enveloped AAV capsid directly mediates many critical host-vector interactions [33]. Until now, more than 100 human and nonhuman primate AAVs have been identified, but only a few, especially those isolated from natural sources, have been applied for human gene transfer [10,33,34]. AAV9, isolated from human liver tissue, can translocate through the blood-brain barrier (BBB), making it a promising gene delivery tool for transducing the central nervous system (CNS) via systemic administration [10,35]. Considering that CNS entry of AAV9 requires a high vector dose, along with the inability of AAV9 to reach all regions of the CNS, there is plenty of room for improving rAAV-based CNS gene delivery [36]. Understanding the molecular mechanism driving AAV9’s CNS entry would enable the development of novel gene delivery systems with enhanced CNS targeting. So far, most identified AAV receptors are glycans [37]. From heparan-sulfate proteoglycans (HSPGs) through sialic acids to terminal galactose moieties, the different AAV serotypes exhibit different carbohydrate specificity [38]. It has been reported that AAV9 binds glycans with terminal galactoses [39,40]. A more recent paper showed that diabetic retinopathy-related retinal changes might improve the transduction efficiency of AAV2 and AAV9 [41]. Enhanced transduction of AAV2 and 9 in diabetic retinopathy is attributed to several overexpressed receptors, such as syndecan-4 (SDC4), glypican-1, and perlecan for AAV2 and 37- and 67-kDa laminin for AAV9. However, the shallow CNS expression of the 37- and 67-kDa laminin receptor contradicts the well-documented preference of AAV9 for adult astrocytes in the CNS [36,42,43,44]. On the other hand, SDC4, the transmembrane proteoglycan overexpressed in diabetic retinopathy, exhibits astrocyte-specific expression in the brain and limited expression in neurons [44,45,46].

Syndecans (SDCs) are a family of evolutionally conserved transmembrane HSPGs sharing a similar structure: a highly conserved, single-pass transmembrane domain and a relatively short cytoplasmic domain [47,48,49,50,51,52]. The more diverse extracellular domain (ectodomain) of SDCs contains glycosaminoglycan (GAG) side chains. SDCs have three GAG attachment sites for HS near the N terminus and may bear chondroitin sulfate (CS) at the juxtamembrane region [49,50,51,52,53]. SDC4 also possesses a cell-binding domain (CBD) mediating cell-to-cell attachment [49,50,51,52]. The versatile GAG chains endow SDCs to interact with many extracellular ligands, including viruses and bacteria [49,50,51,52,54,55,56,57,58]. Besides acting as receptors transmitting extracellular signals into the cell interior, SDCs also facilitate the internalization of their ligands [49,50,51,52,59,60]. SDC isoforms show distinct temporal and spatial expression patterns, thus, are likely to function specifically in vivo [52]. SDC1 is expressed by epithelial and plasma cells, SDC2 by cells of mesenchymal origins (endothelial cells and fibroblasts), SDC3 is enriched in neurons, and SDC4 is more ubiquitous [49,50,51,57,58]. 

Our research group has been characterizing the involvement of SDCs, a conserved family of transmembrane HSPGs, in gene and protein delivery [49,50,51,52,61]. Throughout the years, we have been establishing several SDC-specific assays and SDC structural mutants to analyze the interaction of SDCs with potential ligands [49,50,51,52,57,58,61]. After discovering the involvement of SDC4 in AAV9 cellular entry in human BBB endothelial cells, we applied our previously established and well-characterized SDC assays to understand the contribution of SDCs to AAV9-mediated gene transduction. Utilizing stable transfectants of the various SDC isoforms enabled us to assess the contribution of SDCs’ to AAV9-mediated gene transfer, while studies with structural mutants exposed the involvement of the SDC ectodomain in the interactions with AAV9. Applying quantitative and qualitative cellular assays to explore SDCs’ influence on AAV9-mediated gene delivery helped us to depict a molecularly well-defined mechanism of AAV9 internalization. Our findings thus provide a rational explanation for the low gene delivery potential of AAV9 into neurons in the CNS.

## 2. Results

### 2.1. AAV9 Enters Human BBB Endothelial Cells Attached to SDC4

**Figure 1:**
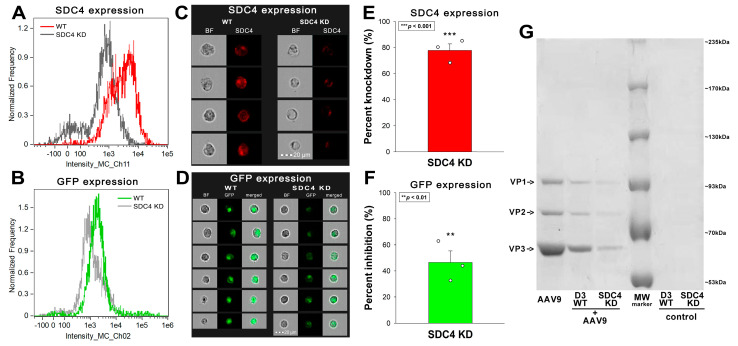
SDC4 contributes to AAV9’s entry into hCMEC/D3 cells. (**A**–**F**) Imaging flow cytometry assessment of SDC4 expression and AAV9-mediated gene (GFP) delivery in wild-type (WT) and SDC4 KD hCMEC/D3 cells. SDC4 KD was performed using SDC4-specific shRNA plasmids. WT and SDC4 KD hCMEC/D3 cells were treated 4 × 10^4^ vg/cell AAV9-GFP for 72 h at 37 °C. SDC4 and GFP expression levels were measured with imaging flow cytometry, as shown by the representative histograms and cellular images of three independent experiments. Scale bar = 20 μm. The effect of SDC4 KD on SDC4 and GFP expression expressed as percent inhibition. The bars represent the mean ± SEM of three independent experiments. Statistical significance vs. WT was assessed with analysis of variance (ANOVA). ** *p* < 0.01; *** *p* < 0.001. (**G**) SDS-PAGE showing VP1-3 immunoprecipitated with SDC4 from AAV9-treated (4 × 10^4^ vg/cell AAv9 for 6 h at 37 °C) hCMEC/D3 cells’ extracts. Lane 1: 10^6^ vg recombinant AAV9; lane 2–3: immunoprecipitates of AAV9-treated WT hCMEC/D3 and SDC4 KD cells; lane 4: MW marker; lane 5–6: immunoprecipitates of WT and SDC4 KD hCMEC/D3 cells untreated with AAV9 (i.e., controls). Standard protein size markers are indicated on the right.

AAV9 is a frequently utilized tool to transduce genes into the CNS. However, the therapeutic application of AAV9 and the development of novel, CNS-specific AAV serotypes are hindered by the limited knowledge of AAV9 receptors, especially those on the BBB. To explore the potential AAV9 receptors on the BBB, we examined AAV9’s entry into hCMEC/D3 cells, a human BBB model cell line with moderate SDC4 expression [62,63]. Efficient SDC4 knockdown significantly reduced AAV9-mediated gene delivery in AAV9-treated (4 × 10^4^ vg/cell at 37 °C for 72 h) hCMEC/D3 cells. Namely, a ~80% reduction in SDC4 expression resulted in a ~50% decrease in AAV9-mediated GFP transduction (Figure 1A–G and Supplementary Figures S1 and S2). The AAV9 capsid proteins (i.e., VP1-3) could be immunoprecipitated with SDC4 from extracts of hCMEC/D3 cells treated with AAV9 (Figure 1G).

**Figure 2:**
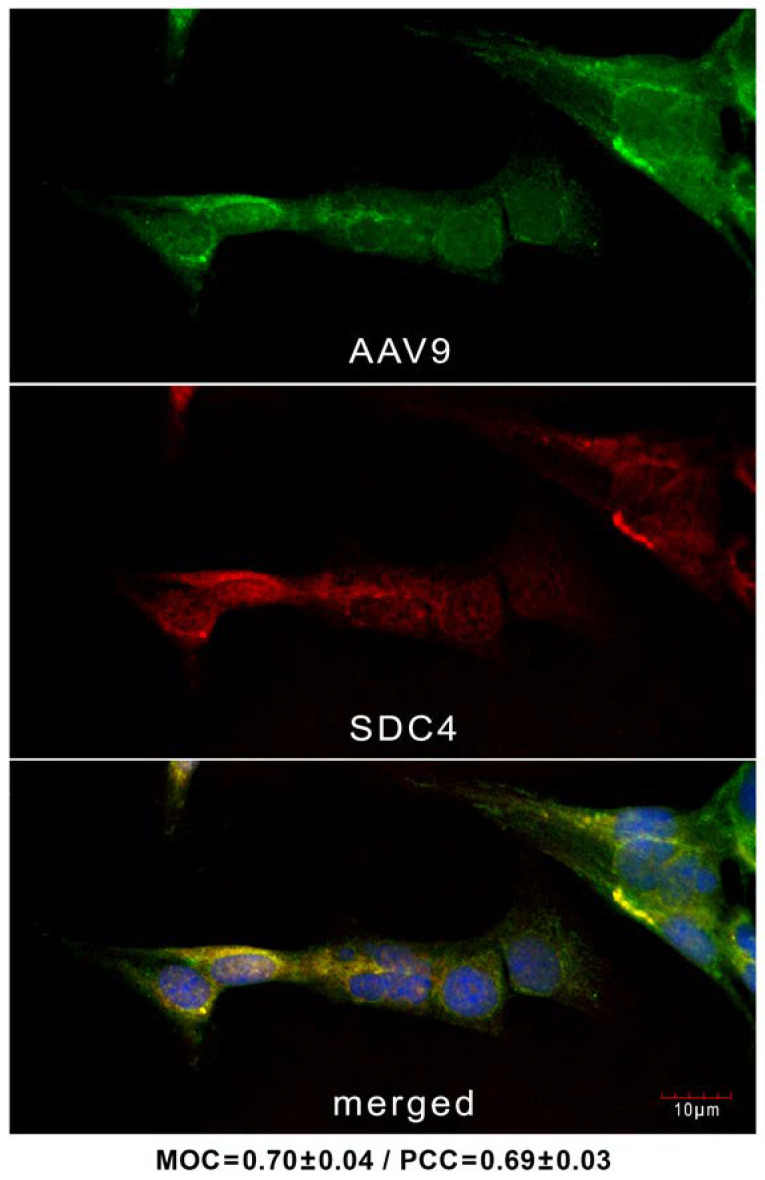
AAV9 colocalizes with SDC4 during cellular internalization. hCMEC/D3 cells were incubated with AAV9 (4 × 10^4^ vg/cell) for 6 h at 37 °C. The cells were then trypsinized, fixed, permeabilized and treated with AF488-labeled AAV9 (green) and APC-labeled SDC4 (red) antibodies. AAV9’s colocalization with SDC4 was analyzed with confocal microscopy. Representative images of three independent experiments are shown. Scale bar = 10 μm. The MOC ± SEM and PCC ± SEM for the overlap and colocalization of SDC4 with AAV9 are indicated below the images.

Confocal microscopic studies on hCMEC/D3 cells treated with AAV9 for 6 h revealed the overlap of AAV9 and SDC4 in AAV9-treated hCMEC/D3 cells, as shown by the high values of the Mander’s overlap and Pearson correlation coefficients (MOC and PCC, respectively) in Figure 2, suggesting that AAV9 enters the cells via an SDC4-mediated pathway. Incubating the cells with the AF 488-labeled secondary antibodies resulted in very low green fluorescence, showing that no unspecific binding influenced the colocalization analyses (Supplementary Figure S3).

### 2.2. Contribution of SDCs to AAV9 Transduction

**Figure 3:**
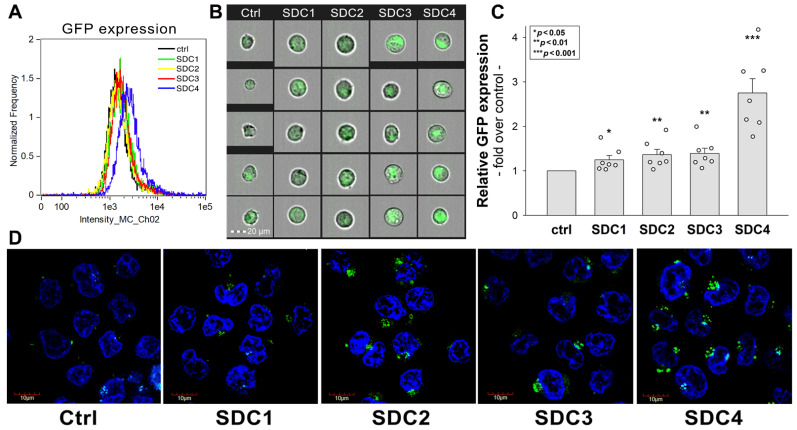
AAV9-mediated GFP transduction into SDC transfectants. WT K562 cells and SDC transfectants were incubated with AAV9-GFP vectors (4 × 10^4^ vg/cell) for 72 h at 37 °C. After incubation, GFP expression was analyzed with imaging flow cytometry and confocal microscopy. (**A***,***B**) Representative flow cytometry histograms and fluorescent images showing the intracellular fluorescence of AAV9-GFP-treated WT K562 cells and SDC transfectants. Scale bar = 20 μm. (**C**) Detected fluorescence intensities were normalized to AAV9-GFP-treated WT K562 cells as standards. The bars represent the mean ± SEM of seven independent experiments. Statistical significance vs. standards was assessed with ANOVA. * *p* < 0.05; ** *p* < 0.01; *** *p* < 0.001. (**D**) Confocal microscopic visualization of AAV9-GFP-treated WT K562 cells and SDC transfectants. Representative images of three independent experiments are shown. Scale bar = 10 μm.

A survey of ex vivo/in vitro transduction efficiency of mammalian cell lines with AAV1-9 showed that none of the AAV serotypes efficiently transduce the human myelogenous leukemia cell line K562 [64]. It is well established that K562 cells lack HSPGs except for minor endogenous betaglycan [65,66]. Due to their very low AAV transducibility and reportedly minimal amount of HSPG expression, K562 cells offer an ideal model to study the effect of SDCs on AAV9-mediated gene delivery [49,50,57,58]. Thus, K562 cells were utilized to create stable SDC transfectants that were standardized according to their HS expression (Supplementary Figure S4) [49,50]. SDC transfectants with a similar level of HS expression were selected and, along with WT K562 cells, transfected with recombinant AAV9 expressing GFP (AAV9-GFP). After 72 h of incubation with AAV9-GFP, GFP expression was examined with imaging flow cytometry and confocal microscopy (Figure 3A–D). Compared to WT K562 cells, overexpression of SDCs resulted in significantly increased AAV9-mediated GFP transduction, as revealed by the significantly higher GFP expression of SDC transfectants (Figure 3A–D). Although SDC1-3 increased AAV9-mediated gene delivery only slightly, the effect of SDC4 overexpression was more profound, increasing AAV9-mediated gene transduction almost threefold (Figure 3A–D). (Here, we have to refer to our previous studies showing that SDC overexpression does not increase the cellular uptake of transferrin, the marker of clathrin-mediated endocytosis [49,50]. These previous studies suggest that SDCs drive an endocytic uptake independent of classical endocytic routes). Co-immunoprecipitation also confirmed that SDCs, especially SDC4, bind AAV9, showing a higher affinity of AAV9 towards SDC4 (Supplementary Figure S6).

### 2.3. Contribution of the SDC4 Ectodomain to AAV9 Uptake

**Figure 4:**
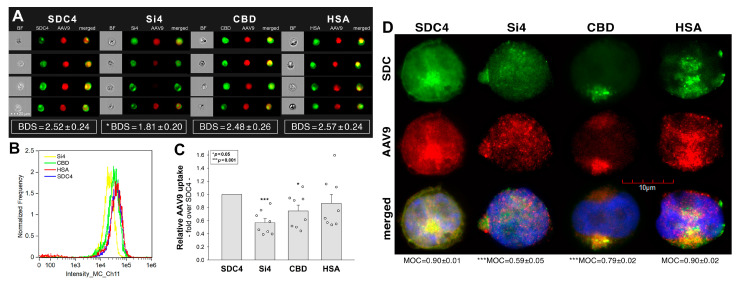
Contribution of the various parts of the SDC4 ectodomain to AAV9 uptake. GFP-tagged SDC4 mutants incubated with AAV9 vectors at 4 × 10^4^ vg/cell for 6 h were fixed, permeabilized, and treated with specific primary AAV9 and AF 633-labeled secondary antibodies. The cellular uptake of AAV9 was then analyzed with imaging flow cytometry and confocal microscopy. (**A**,**B**) Representative fluorescent images and flow cytometry histograms showing the intracellular fluorescence of AAV9-treated SDC4 mutants. Scale bar = 20 μm. The indicated BDS values of AAV9 and SDCs represent the mean + SEM of eight independent experiments. Statistical significance was assessed with ANOVA. (**C**) Detected fluorescence intensities were normalized to AAV9-treated transfectants expressing GFP-labeled WT SDC4 as standards. The bars represent mean + SEM of eight independent experiments. Statistical significance vs. standards was assessed with ANOVA. * *p* < 0.05; *** *p* < 0.001. (**D**) Confocal microscopic visualization of AAV9-treated SDC4 mutants. Scale bar = 10 μm. MOC ± SEM for the overlap of AAV9 with the mutants was calculated by analysis of 15 images with ~10 cells in each image (from three separate samples). Statistical significance vs. AAV9-treated transfectants expressing WT SDC4 (standards) was assessed with ANOVA. *** *p* < 0.001.

Next, we examined the influence of the SDC4 extracellular domain on the interactions with AAV9 by generating various SDC4 deletion mutants (Figure 4A). Si4 is an SDC4 deletion mutant with a truncated ectodomain made of only the signal sequence (Si). The CBD mutant’s extracellular domain is compromised of the signal sequence and the cell-binding domain (CBD) yet lacks the HS attachment site (HSA) and the HS chains (Supplementary Figure S7) [49,50,51,52]. HSA mutants, on the other hand, have an ectodomain made of the HSA and the HS chains but not the CBD [49,50,51,52]. These SDC4 mutants and WT SDC4 were tagged with GFP and expressed in K562 cells (Figure 4A). Clones with an equal extent of SDC expression were then selected with flow cytometry and treated with AAV9. After 90 min of incubation, the AAV9-treated cells were fixed and treated with AAV9-specific and AF 633-labeled secondary antibodies. Cellular fluorescence was then assessed with imaging flow cytometry and confocal microscopy. Based on the detected red (i.e., AF 633) fluorescence intensities, WT SDC4 transfectants internalized AAV9 the most (Figure 4B–D). Deleting both the CBD and the HS chains (i.e., the HSA) exerted a marked reduction in AAV9 uptake as shown by the markedly low fluorescence intensities detected on the Si4 mutants (Figure 4A–D). On the other hand, HS deletion resulted in a much lighter, yet significant, decrease in AAV9 uptake, as demonstrated by the CBD mutants. HSA mutants with an ectodomain made of the HSA and HS chains exhibited an AAV9 uptake efficacy similar to that of SDC4. Colocalization studies also showed a high degree of colocalization between SDC4 and AAV9, as reflected by the high values of bright detail similarity (BDS) and MOC (Figure 4A,D) [67,68]. Deleting both the HSA and the CBD resulted in significantly decreased colocalization. At the same time, deleting either of the CBD or HSA still resulted in high colocalization values (Figure 4A,D), suggesting that both the HS chains and the CBD have essential roles in attaching AAV9. Thus, the CBD can compensate for the loss of HS chains and vice versa.

### 2.4. Effect of Undersulfation on AAV9-Mediated Gene Transduction

**Figure 5:**
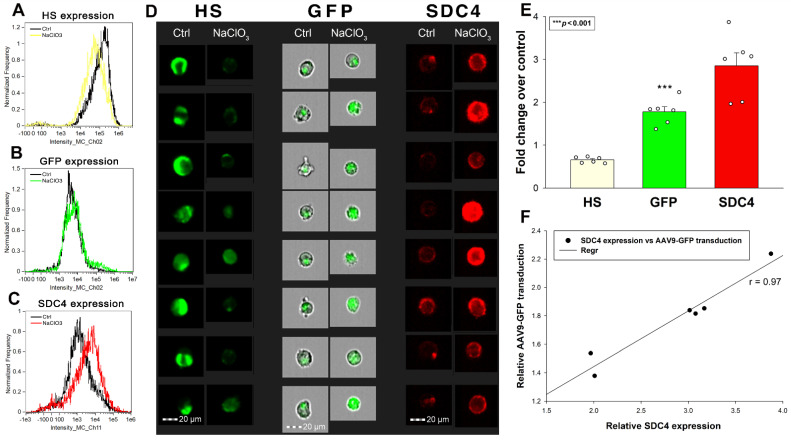
The effect of undersulfation on SDC4 expression and AAV9-mediated gene transduction. Stable SDC4 transfectants (created in K562 cells) were preincubated with or without NaClO_3_ for 48 h. Effect of NaClO_3_ preincubation on HS and SDC4 expression was measured with imaging flow cytometry by incubating the cells with HS- and SDC4-specific antibodies. SDC4 transfectants preincubated with or without NaClO_3_ were then treated with AAV9-GFP vectors at 4 × 10^4^ vg/cell. After 72 h of incubation with AAV9-GFP, GFP expression was measured with imaging flow cytometry. (**A**–**D**) Representative flow cytometry histograms and fluorescent cellular images showing HS, GFP, and SDC4 expression of cells preincubated with or without NaClO_3_. Scale bar = 20 μm. (**E**) Detected HS, GFP, and SDC4 expression levels in SDC4 transfectants were normalized to those untreated with NaClO_3_ (controls). The bars represent the mean + SEM of six independent experiments. Statistical significance was assessed with ANOVA. *** *p* < 0.001. (**F**) Linear regression between SDC4 expression and AAV9-mediated GFP transduction.

The multiple biological activities of HSPGs largely depend on the interactions of their HS chains with various ligands [69]. To further investigate the contribution of SDCs’ HS chains to the interactions with AAV9, we induced undersulfation of SDC4 transfectants with sodium chlorate (NaClO_3_), an inhibitor of proteoglycan sulfation, and measured its effect on AAV9-mediated gene transduction [70]. Initial studies delivered surprising results: although NaClO_3_ reduced HS expression, AAV9-mediated GFP expression increased (Figure 5A,B,D,E). Investigating these contradictory results, we found that SDC4 expression also increased (almost tripled) after 48 h of NaClO_3_ treatment (Figure 5C–E). Thus, SDC4 transfectants compensate for the loss of HS by increasing SDC4 expression. Although the increased SDC4 expression is associated with reduced HS content, AAV9-mediated gene delivery increases due to the high binding of AAV9 to the SDC4 core protein. Thus, SDC4 correlates with AAV9-mediated gene delivery, regardless of the HS content of SDC4 (Figure 5F). Undersulfation with NaClO_3_, therefore, confirmed previous studies with SDC4 mutants, namely that the SDC4 core protein, including the CBD mediating cell to cell attachment, also takes part in attaching AAV9.

### 2.5. Affinity Proteomics Exploration of AAV9 Interactome

After demonstrating SDC4’s correlation with AAV9-mediated gene transduction, we examined the AAV9 interactome in SDC4 transfectants created in K562 cells (Table 1). The affinity-based pull-down experiments unraveled a viral entry route that can be characterized by proteins involved in the SDC4 endocytic pathway, including Rab7 (P51149) small GTPase, VAMP8 (Q9BV40), and perilipin-3 (O60664). The virus RNA synthesis is most probably increased by CDC11 (P21127) and CDC12 (J3QSD7) phosphorylating the CTD of RNA Pol. II. The mRNAs, which code the virus proteins, are transported via the TREX-THOC complex (THO-1 (Q96FV9), -2 (Q8NI27), -4 (Q86V81)) from the nucleus. The virus particle secreted by sec23A (Q15436) is a part of COPII complex, perilipin-3 (O60664), Rab1b (Q9H0U4), and Rab11 (P62491) pathway. Rab1b was also reported in the secretion of HCV [71]. AAV9 can also bind to the desmosomes, as desmoplakin (P15924) and desmoglein (Q02413) were pulled down along with the cytokeratins Keratin type I (Q7Z3Y8) and II (F8VV57).

### 2.6. Effect of SDC4 Knock-In in Neuronal Cells

**Figure 6:**
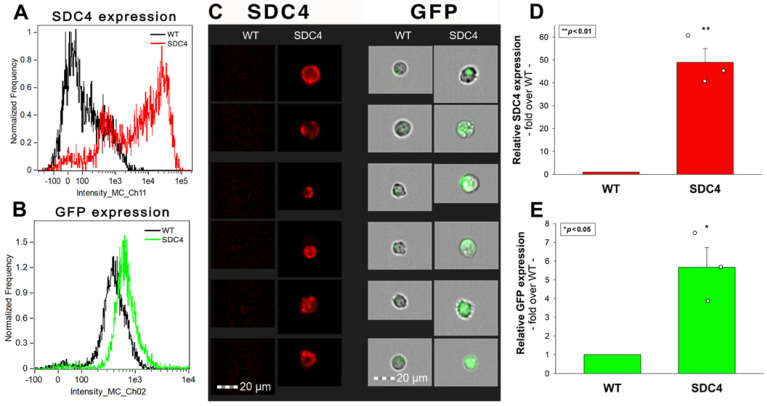
SDC4 overexpression increases AAV9-mediated GFP transduction in SH-SY5Y cells. SDC4 transfectants (created in SH-SY5Y cells) and WT SH-SY5Y cells were incubated with AAV9-GFP (4 × 10^4^ vg/cell) at 37 °C for 72 h. GFP expression was then analyzed with imaging flow cytometry. (**A**–**C**) Representative flow cytometry histograms and fluorescent images showing the GFP and SDC4 expression levels in WT SH-SY5Y cells and SDC4 transfectants treated with AAV9-GFP. Scale bar = 20 μm. (**D**,**E**) Detected SDC4 and GFP expression levels of AAV9-treated SDC4 transfectants were normalized to that of WT SH-SY5Y cells as standards. The bars represent the mean + SEM of three independent experiments. Statistical significance vs. standards was assessed with ANOVA. * *p* < 0.05; ** *p* < 0.01.

In the CNS, the AAV9 has an affinity towards astrocytes while exhibiting limited neuronal transduction [36,42,43]. SH-SY5Y cells, a frequently utilized neuroblastoma cell line, show shallow levels of SDC4 expression (Figure 6A,C). Overexpression of SDC4 in SH-SY5Y transfection induced a marked increase in AAV9-mediated GFP transduction, as detected with imaging flow cytometry (Figure 6A–E).

### 2.7. Glial Cells Could Be Transduced More Efficiently with AAV9 than Neuron-like Cells

**Figure 7:**
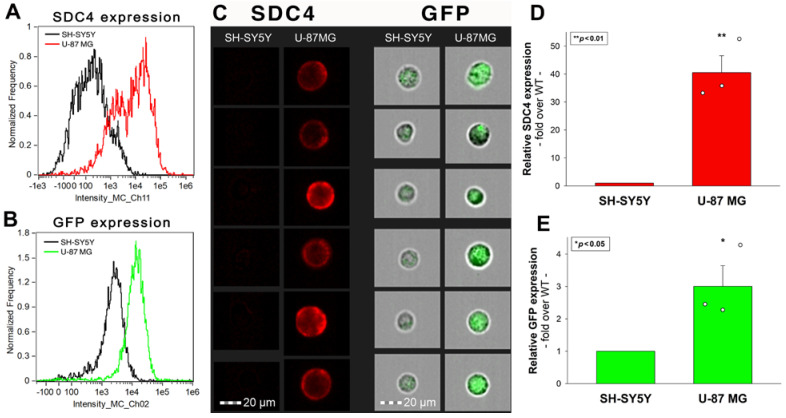
AAV9-mediated GFP transduction is more efficient in U-87 MG than in SH-SY5Y cells. WT U-87 MG and SH-SY5Y cells were incubated with AAV9-GFP (4 × 10^4^ vg/cell) for 72 h. GFP expression was then analyzed with imaging flow cytometry. (**A**–**C**) Representative flow cytometry histograms and fluorescent images showing the SDC4 and GFP expression levels in SH-SY5Y and U-87 MG cells treated with AAV9-GFP. Scale bar = 20 μm. (**D**,**E**) Detected SDC4 and GFP expression levels of AAV9-treated U-87 MG cells were normalized to that of SH-SY5Y cells as standards. The bars represent the mean + SEM of three independent experiments. Statistical significance vs. standards was assessed with ANOVA. * *p* < 0.05; ** *p* < 0.01.

U-87 MG is a glial (i.e., glioblastoma) cell line exhibiting significantly (~40×) higher SDC4 expression than the neuronal-like (i.e., neuroblastoma) SH-SY5Y cells with shallow SDC4 expression (Figure 7A,C,D). Treatment with recombinant AAV9-GFP vectors resulted in markedly (~3×) increased GFP expression in SDC4-enriched U-87 MG cells than in SH-SY5Y cells with minimal SDC4 expression, providing further evidence of the involvement of SDC4 in mediating AAV9 cellular entry (Figure 7B,C,E).

## 3. Discussion

AAV9 is a promising viral vector to deliver genes into the CNS. Given the genetic backgrounds of several CNS disorders, targeted manipulation of CNS gene expression offers new therapeutic opportunities for CNS diseases with unmet medical needs. Breakthrough therapeutic innovations for CNS disorders are still waiting to be achieved. The still unknown details of AAV9 cellular biology detain the successful therapeutic implementation of AAV9-based CNS therapies.

After peripheral (i.e., intravenous) administration, AAV9 induces robust gene expression throughout the mice CNS [44]. The pattern of gene delivery differs with age: neonatal subjects exhibit greater neuronal transduction than adults, which predominantly show astroglial versus neuronal transduction [36]. The different membrane expression profiles of astroglia and neurons must account for AAV9’s more efficient astroglial transducibility. However, the molecular mechanisms accounting for AAV9’s astroglial preference are still not fully understood. Thus, the incomplete understanding of AAV9 cellular receptors greatly hampers the targeted application of AAV9-based CNS gene therapies.

To find answers to the unknown details of AAV9-mediated gene delivery, we decided to explore the potential BBB receptors of AAV9. Studies on AAV9-treated human BBB endothelial cells showed that knockdown of SDC4, the universally expressed member of the SDC family of transmembrane HSPGs, decreased AAV9-mediated GFP transduction. SDC-specific cellular models created in poorly transducible K562 cells showed that among SDCs, SDC4 is the major facilitator of AAV9’s cell entry. Overexpression of all SDC isoforms, including the epithelial SDC1, the endothelial SDC2, and the neuronal SDC3, increased AAV9-mediated gene delivery. However, the most significant increase in AAV-mediated GFP transduction was due to SDC4 overexpression. AAV9’s interaction with SDC4 involved the HS chains and the CBD domain of the SDC4 ectodomain, highlighting also the importance of GAG-independent constituents of the SDC4 core protein in attaching AAV9. The reduction in the cellular entry of AAV9 after the removal of SDC4’s HS chains was reasonably low, while overexpressing SDC4 with reduced HS chains still increased AAV9-mediated gene delivery. These results showed that CBD of SDC4 also binds AAV9. Affinity proteomics on SDC4 transfectants revealed that AAV9 interacts with SDC4’s intracellular partners. Overexpression of SDC4 into the poorly transducible neuroblastoma SH-SY5Y cell line increased AAV9-mediated gene delivery. The U-87 MG glial cell line with high SDC4 expression could also be transduced more efficiently than SH-SY5Y cells, serving evidence on the more efficient transducibility of SDC4-enriched glial cells than neurons with modest SDC4 expression.

In summary, our data provides detailed cellular evidence of SDC4’s involvement in the cellular internalization of AAV9. The establishment of SDC4 as a significant mediator of AAV9 entry also provides a molecularly valid explanation of AAV9’s astrocyte preference in adult mice brains. It is worth noting that in rodents, SDC4 expression is typical of astroglia, the main cellular targets of AAV9 in the CNS of adult mice [36,44,45,72]. Exploiting the described data could thus provide more rational strategies for developing targeted AAV9-based gene therapies.

## 4. Materials and Methods

### 4.1. Recombinant AAV9 Vectors

The N-terminally AAV9 expressing green fluorescent protein (GFP) under the control of the chicken-β-actin hybrid promoter was produced as described previously [73].

### 4.2. Establishment of SDC4 KD Cell Lines

SDC4 knockdown in hCMEC/D3 was performed using a SDC4 shRNA plasmid (cat. no. sc-36588-SH), using shRNA plasmid transfection reagent (cat. no. sc-108061) according to the manufacturer’s protocol (Santa Cruz Biotechnology, Inc., Dallas, TX, USA). Control hCMEC/D3 cells used in the study were indeed treated with control for shRNA plasmids, scrambled shRNA sequences that did not lead to the specific degradation of SDC4 or any known cellular mRNAs (cat. no. sc-108060, Santa Cruz Biotechnology, Inc., Dallas, TX, USA). Stable KD cells were selected in 2 mg G418 and sorted using imaging flow cytometry (Amnis® FlowSight®, Luminex Corporation, Austin, TX, USA) with APC-conjugated anti-SDC4 antibody (RnD Systems, Minneapolis, MN, USA, cat. no. FAB29181A, 5:100 dilution) and respective isotype control (rat IgG2A APC isotype control, RnD Systems, cat. no. IC006A, 5:100). The cellular expression of SDC4 following knockdown was also determined with Western blotting as described previously [74]. A chemiluminescence detection reagent (Luminata Crescendo Western Blotting HRP Reagents) was used for protein visualization, and the signal was detected with UVITEC Alliance Q9 Advanced imager (Uvitec Ltd., Cambridge, UK). β-Tubulin (mouse monoclonal, Santa Cruz Biotechnology Inc., Dallas, TX, USA, cat. no. sc-5274) was used as a loading control (in 1:100 dilution).

### 4.3. SDC Constructs, Cell Culture and Transfection

Full-length SDC1-4 and SDC4 deletion mutants and transfectants, established in K562 (ATCC^®®®^ CCL-243™) and SH-SY5Y cells (ATCC^®®®^ CRL-2266™), were created as described previously [49,50,51]. Stable SDC transfectants were selected by measuring SDC expression with flow cytometry using APC-labeled SDC antibodies specific for the respective SDC isoform (all RnD Systems, Minneapolis, MN, USA; SDC1: monoclonal rat IgG1 Clone #359103, cat. no. FAB2780A; SDC2: monoclonal rat IgG2B Clone #305515, cat. no. FAB2965A [74,75,76]; SDC3: polyclonal goat IgG, cat. no. FAB3539A [74,77]; SDC4: monoclonal rat IgG2a clone #336304, cat. no. FAB29181A [49,50]), along with respective isotype controls (all RnD Systems; rat IgG1 APC-conjugated isotype control, cat. no. IC005A; rat IgG2B APC-conjugated isotype control, cat. no. IC013A; goat IgG APC-conjugated antibody, cat. no. IC108A; rat IgG2A APC isotype control, cat. no. IC006A).

### 4.4. Flow Cytometry Analysis of HS and SDC Expression

HS and SDC expression of the applied cell lines (hCMEC/D3, K562, and SDC transfectants, U87 MG and SH-SY5Y cell) were measured with flow cytometry by using anti-human HS antibody (10E4 epitope; Amsbio, Abingdon, UK; with Alexa Fluor [AF] 647-labeled secondary anti-mouse IgM and respective isotype control: Thermo Fisher Scientific, Waltham, MA, USA, cat. no. 02-6800) and APC-labeled SDC antibodies as described previously [49,50]. SDC transfectants with almost equal amounts of HS expression were selected for further uptake studies [49,50].

### 4.5. Flow Cytometry Analysis of AAV9-Mediated GFP Transduction and AAV9 Uptake

WT K562, SH-SY5Y, U-87 MG, WT and SDC4 KD hCMEC/D3 cells, SDC transfectants and SDC4 mutants were utilized to quantify AAV9-mediated GFP transduction. Briefly, 6 × 10^5^ cells/mL in DMEM/F12 medium were incubated with AAV9-GFP vectors at 4 × 10^4^ vg/cell. GFP expression was analyzed 72 h later with imaging flow cytometry (Amnis® FlowSight®, Luminex Corporation, Austin, TX, USA). In the case of SDC4 mutants, after 6 h of incubation with AAV9, the cells were trypsinized (with the method described by Nakase et al. [78]) to remove the extracellularly attached virus particles. The cells were then treated with mouse monoclonal (ADK9) to AAV9 (1:100 dilution, Progen, Wayne, PA, USA, cat.no: 690162), followed by treatment with AF 633-labeled goat anti-mouse IgG (1:100 dilution, Invitrogen A-21052, Waltham, MA, USA). The samples were then rinsed three times with PBS containing 1% BSA and 0.1% Triton X-100 and progressed toward flow cytometry. Cellular uptake was then measured with flow cytometry using an AMNIS® FlowSight imaging flow cytometer (AMNIS Corporation, Seattle, WA, USA). A minimum of 5000 events per sample was analyzed. Laser power was identical within each experiment. Appropriate gating in a forward-scatter-against-side-scatter plot was utilized to exclude cellular debris and aggregates. Fluorescence analysis was conducted with the IDEAS™ analysis software. To examine the influence of the fluorescently labeled secondary IgGs, some cells were also treated with secondary antibodies without AAV9 treatment or preincubation with primary anti-AAV9 antibodies.

### 4.6. Microscopic Visualization of Uptake

AAV9’s cellular entry and AAV9-mediated GFP transduction were visualized with confocal microscopy. hCMEC/D3 cells, SDC transfectants, and SDC4 mutants (established in WT K562 cells) were grown on poly-D-lysine-coated glass-bottom 35-mm culture dishes (MatTek Corp., Ashland, MA, USA). The cells were preincubated in DMEM/F12 medium at 37 °C for 24 h before incubation with the AAV9-GFP at a concentration of 4 × 10^4^ vg/cell for 72 h at 37 °C. For colocalization studies, hCMEC/D3 cells and SDC4 mutants were incubated with AAV9-GFP for 6 h at 37 °C. Then cells were rinsed two times with ice-cold PBS and fixed in 4% paraformaldehyde (Sigma, St. Louis, MO, USA); the cell membranes were permeabilized (0.1% Triton X-100), and the cells were blocked with the appropriate serum for 1h at room temperature, followed by the specific antibody treatments as described for the flow cytometry analyses. The samples were then rinsed three times with PBS containing 0.1% Triton X-100, then stained with DAPI (1:5000) for 5 min, washed three times with PBS, and embedded in Fluoromount G (SouthernBiotech, Birmingham, AL, USA) [49,50]. The fluorescence distribution was then analyzed on a Leica DMi8 microscope equipped with Aurox Clarity Laser Free Confocal Unit. Sections presented were taken approximately at the mid-height level of the cells. Photomultiplier gain and illumination power were identical within each experiment. Aurox Visionary software was used for image acquisition by confocal microscopy. For colocalization analyses, the images were analyzed with ImageJ’s (NIH, Bethesda, MD, USA) JACoP plugin [79]. AAV9-mediated GFP transduction into WT K562 cells and SDC transfectants was then visualized with an Olympus FV1000 confocal laser scanning microscope equipped with three lasers. A laser diode (excitation, 405 nm) and a band-pass filter (420–480 nm) were used to capture the signal recorded as blue; an argon laser (excitation, 488 nm) and a band-pass filter (505–530 nm) were used to capture the signal recorded as green; and, finally, a helium/neon laser (excitation, 543 nm) and a band-pass filter (550–625 nm) were used to capture the signal recorded as red. Sections presented were taken approximately at the mid-height level of the cells. Photomultiplier gain and laser power were identical within each experiment. The Olympus Fluoview software (version 4.2b) was used for image acquisition and analysis.

### 4.7. Undersulfation Studies

Stable SDC4 transfectants (created in K562 cells) were preincubated with or without 60 mM sodium chlorate (NaClO_3_; Sigma, St. Louis, MO, USA) for 48 h. The effect of NaClO_3_ preincubation on HS and SDC4 expression was measured with imaging flow cytometry by incubating the cells with HS- and SDC4-specific antibodies. SDC4 transfectants preincubated with or without NaClO_3_ were then treated with AAV9-GFP vectors at 4 × 10^4^ vg/cell. After 72 h of incubation with AAV9-GFP, GFP expression was measured with imaging flow cytometric analyses described above.

### 4.8. Co-Immunoprecipitation Experiments

WT and SDC4 KD hCMEC/D3 cells, WT K562, and SDC transfectants incubated with or without AAV9 were processed for co-immunoprecipitation experiments as described previously [49,50]. Briefly, after incubation, the cells were washed twice with ice-cold PBS and treated with a cold Pierce IP lysis buffer. The cells were then scraped off to clean Eppendorf tubes, put on a low-speed rotating shaker for 15 min, and centrifuged at 14,000× *g* for 15 min at 4 °C. The supernatant was then transferred to new tubes and combined with 5 µg human SDC4 antibody (RnD System, Abingdon, UK; cat. no. AF2918). The antigen sample/SDC antibody mixture was then incubated overnight at 4 °C with mixing. The antigen sample/antibody mixture was then added to a 1.5 mL microcentrifuge tube containing pre-washed Pierce Protein A/G Magnetic Beads (Thermo Fisher Scientific, Waltham, MA, USA). After incubation at room temperature for 1 h with mixing, the beads were then collected with a MagJET Separation Rack magnetic stand (Thermo Fisher Scientific, Waltham, MA, USA), and supernatants were discarded. To elute the antigen, 100 µL of SDS-PAGE reducing sample buffer was added to the tubes. The samples were heated at 96 °C for 10 min in 1% SDS and transferred to SDS-PAGE [49,50]. The samples were then immunoblotted onto PVDF membranes, and the proteins were detected with specific antibodies, as described above. Image acquisition was conducted with UVITEC Alliance Q9 Advanced imaging platform.

### 4.9. Affinity-Based Proteomics of SDC4 Interactions in SDC4 Transfectants

To reveal the AAV9 interactome, whole cell extracts were prepared from a stable SDC4 transfectant. Then an AAV9 co-immunoprecipitation (AAV9 co-IP) assay (using mouse monoclonal antibody (ADK9) to AAV9 (1:100 dilution, Progen, Wayne, PA, USA, cat.no: 690162)) and subsequent LC-MS/MS analysis using a nanoflow RP-HPLC online coupled to a linear ion trap-Orbitrap (Orbitrap-Elite, Thermo Fisher Scientific, Dreieich, Germany) mass spectrometer was performed as described previously [50]. Raw data were converted into peak lists using Proteome Discoverer (v 1.4, Thermo Fisher Scientific, Waltham, MA, USA). First, we searched against the Swissprot and Uniprot databases, considering the sequence of SDC4. Search parameters and acceptance criteria were set as previously published. Close homologs were only reported if at least three unique peptides matched the protein. Spectral counting was used to estimate the relative abundance of individual proteins in the samples: peptide counts of the individual proteins were normalized to the total number of peptide identifications in each sample [80]. Proteins (i) with reproducible detection (|log2fold-change| < 0.67 between biological replicates), (ii) with at least two identified peptides, (iii) with at least 5% coverage, and (iv) with a median-normalized protein binding affinity score above a previously defined cut-off value were considered as proteins that specifically associate with the AAV9 capsid [81].

### 4.10. Statistical Analysis

Results are expressed as means + standard error of the mean (SEM). Differences between experimental groups were evaluated by using one-way analysis of variance (ANOVA). Values of *p* < 0.05 were accepted as significant [49,50]. During imaging flow cytometry, the bright detail similarity (BDS) feature of the Amnis® IDEAS software was used to measure colocalization between two signals [67]. A BDS score of 2 or greater represents a high degree of overlap.

## Figures and Tables

**Table 1:** The AAV9 interactome in SDC4 transfectants.

UniProt	Number Unique	Peptide Count	RelativePeptide Count	% Coverage	MW	Protein Name
P31431	4	7	0.109	19.2	21,641.7	Syndecan-4
P26038	12	27	1.286	23.9	67,820.6	Moesin
P18206	5	6	0.286	7.1	123,800.4	Vinculin
Q9H0U4	11	21	0.778	55.7	22,171.4	Ras-related protein Rab-1B
P51149	6	14	0.667	38.2	23,490	Ras-related protein Rab-7a
P62491	7	14	0.667	34.3	24,393.7	Ras-related protein Rab-11A
Q96FV9	3	6	0.286	9.4	75,667	THO complex subunit 1
Q8NI27	9	15	0.714	7.7	182,776.2	THO complex subunit 2
Q86V81	5	9	0.429	36.6	26,888.1	THO complex subunit 4
O43290	30	64	2.370	43.2	90,255.5	U4/U6.U5 tri-snRNP-associated protein 1
Q8WVK2	16	64	3.048	39.4	18,860.3	U4/U6.U5 small nuclear ribonucleoprotein 27 kDa protein
Q9BV40	3	4	0.190	38	11,438.4	Vesicle-associated membrane protein 8
Q13838	5	8	0.381	16.8	48,991.8	Spliceosome RNA helicase DDX39B
Q53FV3	3	3	0.143	9.6	46,268.3	COP9 signalosome subunit 4 variant (Fragment)
P26583	9	26	1.238	23.3	24,894	High mobility group protein B1
Q15436	3	4	0.667	4.8	86,161.6	Protein transport protein Sec23A
P16949	4	9	1.500	24.8	17,302.7	Stathmin
A0A024R210	3	4	0.667	16.8	13,938.6	Interferon-induced transmembrane protein 1 (9–27), isoform CRA_a
P17096	5	34	5.667	41.1	11,676.1	High mobility group protein HMG-I/HMG-Y
Q7Z3Y8	3	33	0.009	3.9	49,822.8	Keratin, type I cytoskeletal 27
F8VV57	4	13	0.004	26.5	12,157.3	Keratin, type II cytoskeletal 5 (Fragment)
Q02413	12	21	1.000	12	113,748.6	Desmoglein-1: desmosome resident
P15924	11	15	0.714	5.2	331,776.7	Desmoplakin

## Data Availability

Data are contained within the article or Supplementary Materials.

## Supplementary Materials

The following supporting information can be downloaded at: https://www.mdpi.com/article/10.3390/ijms24043141/s1.
