# Supplementary material for: Syndecan-4 Mediates the Cellular Entry of Adeno-Associated Virus 9
Source: Int J Mol Sci. 2023 Feb 5;24(4):3141. doi: 10.3390/ijms24043141 (PMC9963952; doi:10.3390/ijms24043141)
Supplement: Supplementary file 1 [file ijms-24-03141-s001.zip › ijms-2179255-supplementary.pdf]

Supplementary Materials: Syndecan-4 Mediates The Cellular Entry of Adeno-associated Virus 9

Anett Hudák [1], Matthew Roach [2], Pusztai Dávid [1], Pettkó-Szandtner Aladár [3], Annamária Letoha [4], László Szilák [5], Mimoun Azzouz [6], and Tamás Letoha [1,*]

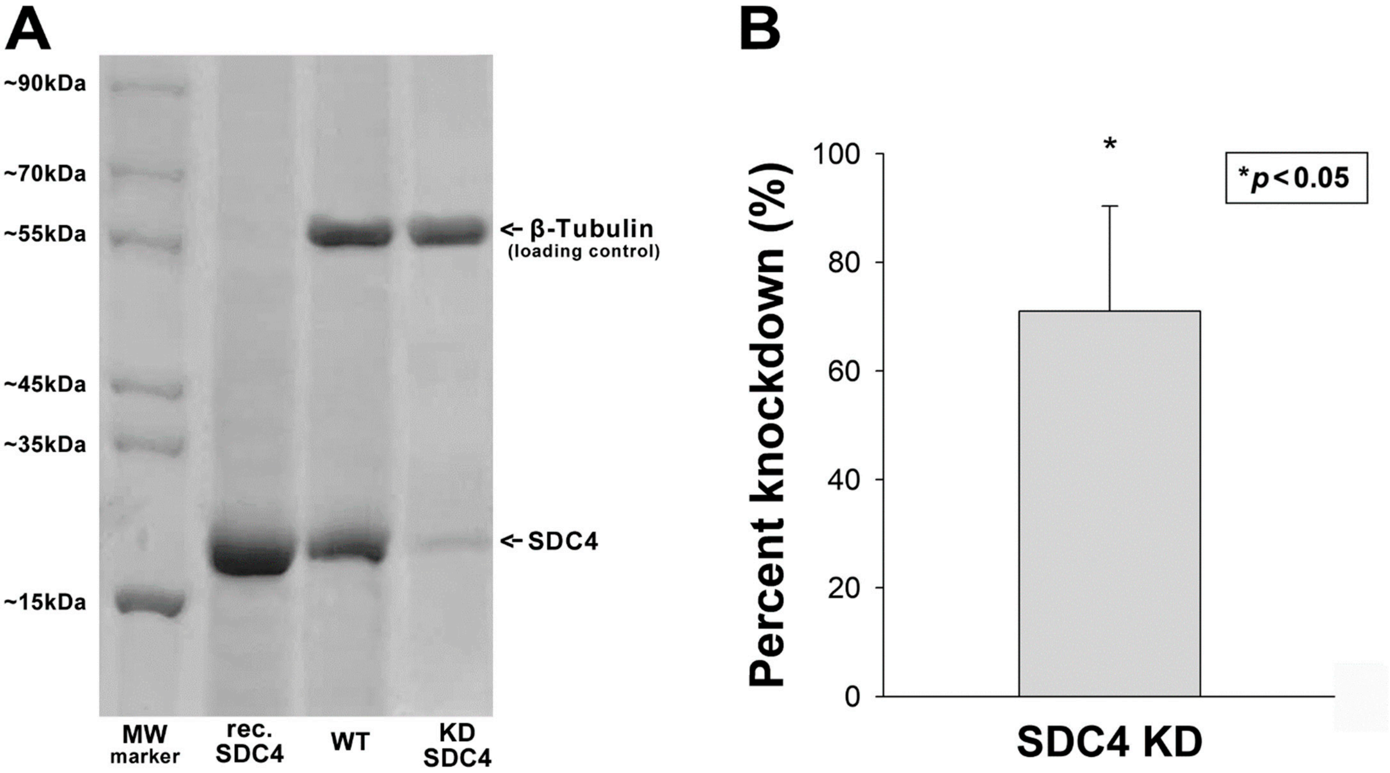

Figure S1. Western blot validation of SDC4 knockdown in hCMEC/D3 cells. SDC4 knockdown in hCMEC/D3 cells was performed using a lentiviral vector system specific to human SDC4 shRNA. Stable KD cells were selected in 2 mg G418 and sorted using imaging flow cytometry (Amnis FlowSight) with APC-conjugated anti-SDC4 antibodies. (**A**) Cellular expression of SDC4 following knockdown was also determined with Western blotting. WT hCMEC/D3 and SDC4 shRNA-treated (i.e., SDC4 KD) cells were grown in 24-well plates for 24 h, then the medium was removed, and the cells were washed (with PBS) and lysed in RIPA buffer. Protein concentrations were measured with a spectrophotometer (Metertech UV/VIS). Equal amounts of protein from cell lysates were then subjected to SDS-PAGE on 7,5%–12,5% gradient gels and electroblotted onto PVDF membranes using the Mini Wide Vertical Electrophoresis gel system (Cleaver Scientific). The membranes were blocked in TBST with 5% dry milk, washed, incubated with anti-SDC4 antibodies diluted in TBST with 1% dry milk for 2 h, and then incubated with HRP-conjugated secondary antibodies. A chemiluminescence detection reagent was used for protein visualization and the signal was detected with UVITEC Alliance Q9 Advanced Imager. β-Tubulin was used as a loading control. (**B**) Detected band intensities were normalized to WT hCMEC/D3 cells as standard. The bars represent the mean + SEM of three independent experiments. Statistical significance vs. standards was assessed with ANOVA. * $p < 0.05$

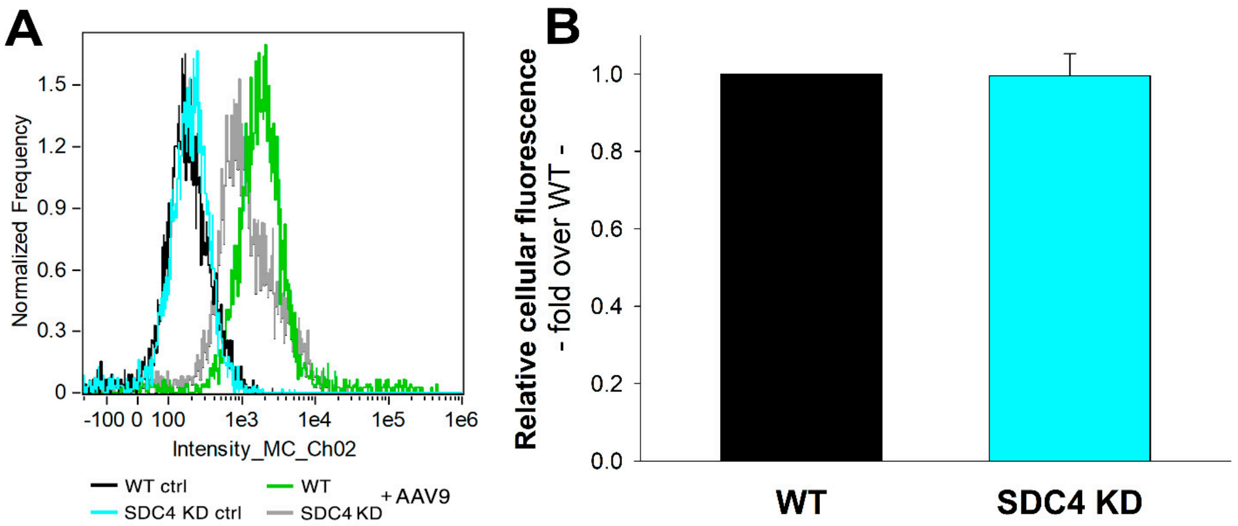

Figure S2. Green autofluorescence of WT and SDC4 KD hCMEC/D3 cells as measured with flow cytometry. **(A)** Representative flow cytometry histogram showing the fluorescence AAV9-treated and untreated (i.e., controls) WT and SDC4 KD hCMEC/D3 cells. (**B**) Detected fluorescence intensities of AAV9-untreated SDC4 KD hCMEC/D3 cells (i.e., SDC4 KD ctrl) were normalized to AAV9-untreated WT hCMEC/D3 cells (i.e., WT ctrl) as standards. The bars represent the mean + SEM of three independent experiments. Statistical significance was assessed with analysis of variance (ANOVA). Compared to standards, no statistical significance was detected.

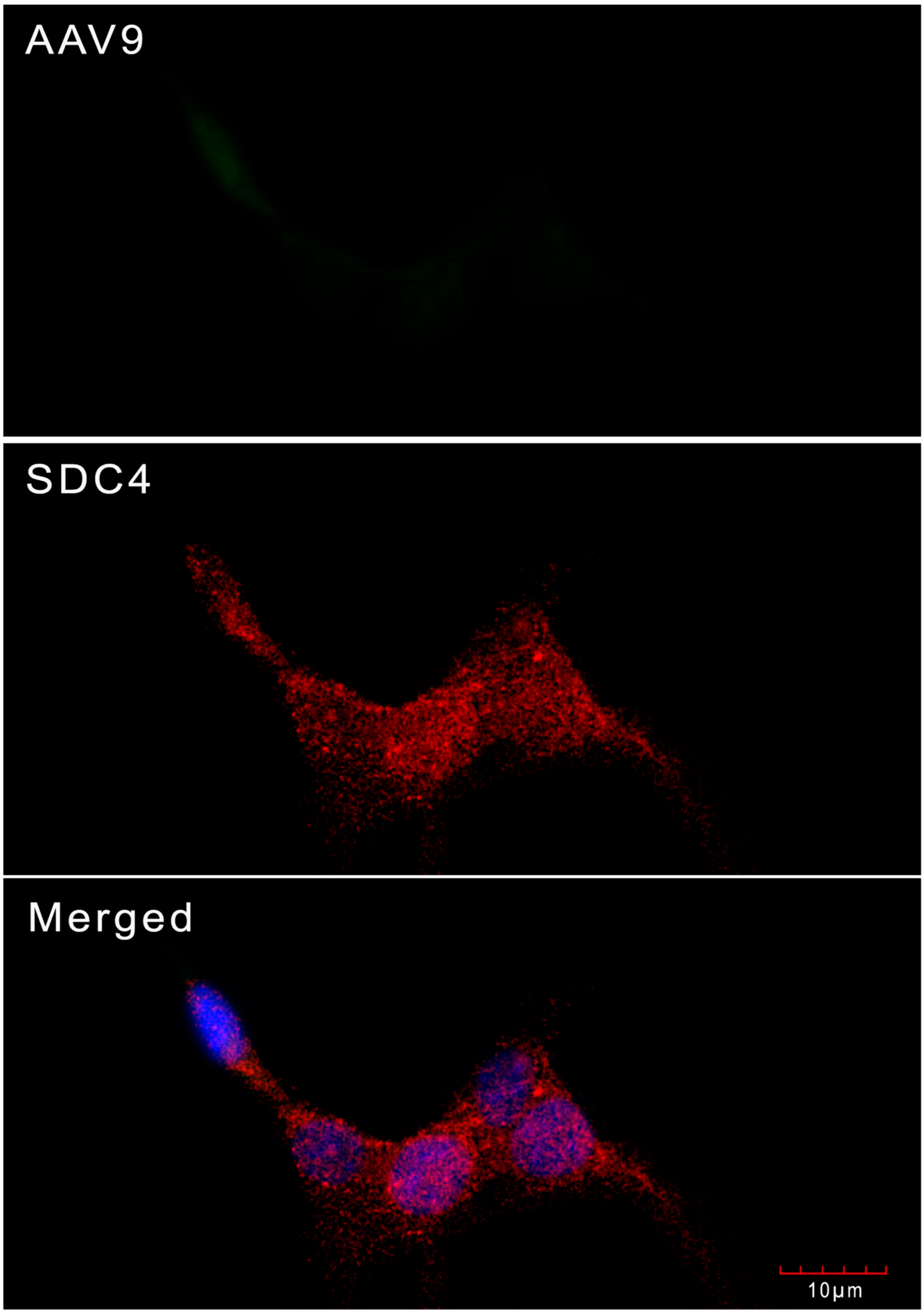

Figure S3. Control colocalization studies on AAV9-treated hCMEC/D3 cells. hCMEC/D3 cells were incubated with AAV9 (4 x 10^4 vg/cell) for 6h at 37 °C. The cells were then trypsinized, fixed, permeabilized, and treated with AF488-labeled anti-mouse secondary and APC-labeled SDC4 (red) antibodies. Representative images of three independent experiments are shown. Scale bar = 10 µm. MOC and PCC values for the overlap and colocalization of SDC with AAV9 are indicated below the images.

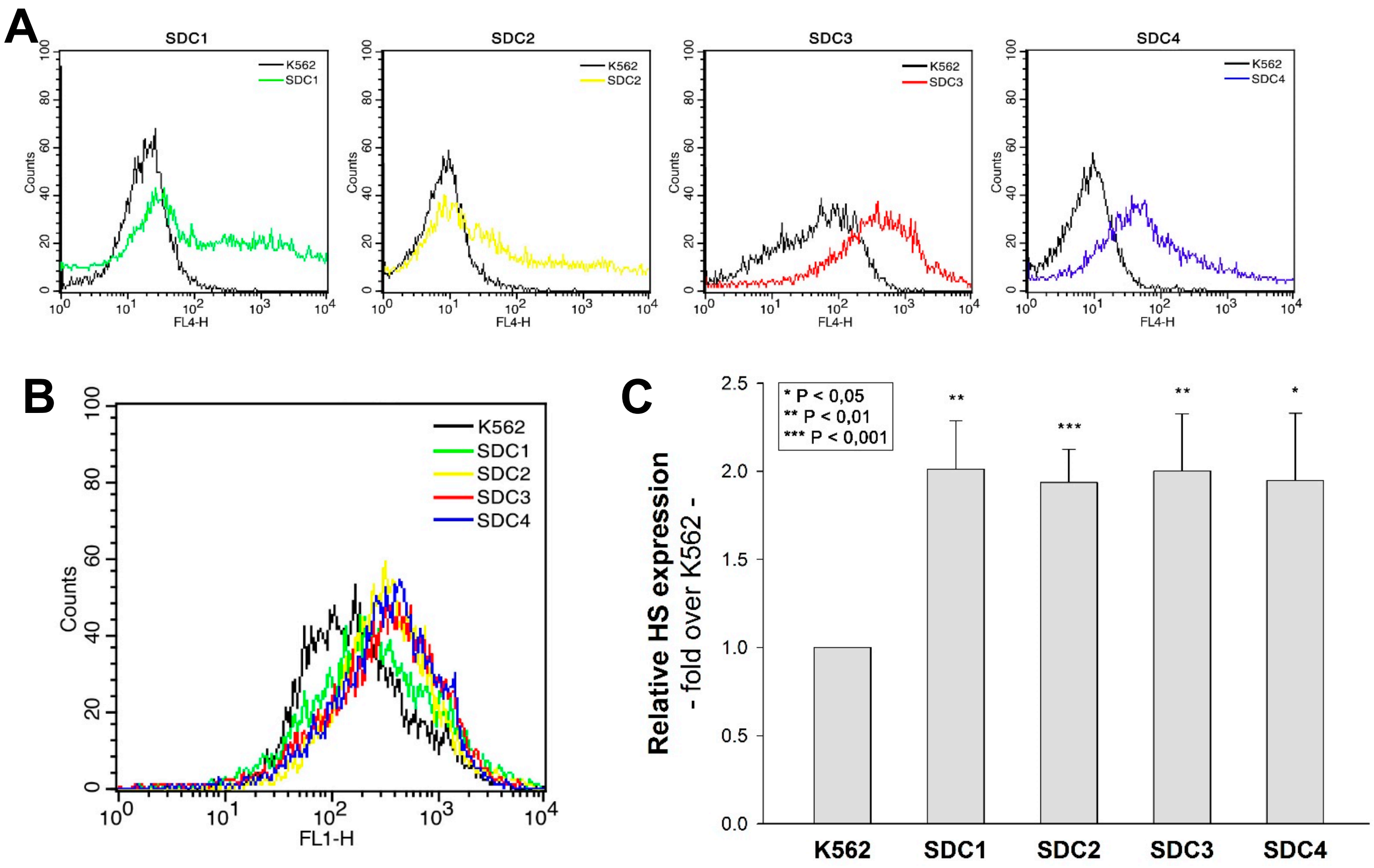

Figure S4. Relative HS expression of SDC transfectants. (**A**) Stable SDC transfectants created in wild-type (WT) K562 cells were selected by measuring SDC expression with flow cytometry (Becton Dickinson FACScan) using APC-labeled anti-SDC antibodies specific for each SDC isoform. (**B**) Flow cytometry histograms showing HS expression of SDC transfectants and WT K562 cells. HS expression of SDC transfectants, along with WT K562 cells, was measured by flow cytometry (Becton Dickinson FACScan) using an anti-HS antibody. SDC transfectants with similar HS expression were selected and applied for further studies. (**C**) Detected HS levels were normalized to WT K562 cells as standards. The bars represent the mean + SEM of ten independent experiments. Statistical significance vs. WT K562 cells (standards) was assessed with analysis of variance (ANOVA). *$p<0.05$ vs WT K562 cells; **$p<0.01$ vs WT K562 cells, ***$p<0.001$ vs WT K562 cells.

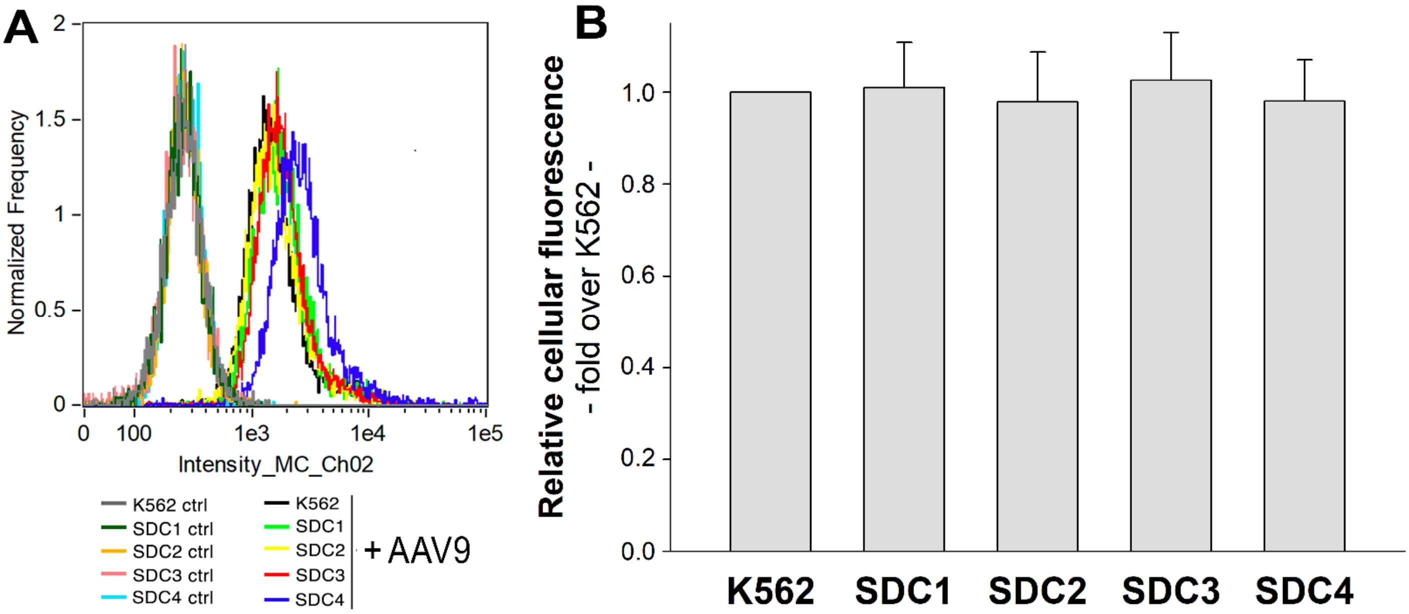

Figure S5. Green autofluorescence of WT K562 cells and SDC transfectants as measured with flow cytometry. (**A**) Representative flow cytometry histogram showing the fluorescence AAV9-treated and untreated (i.e., controls) WT K562 cells and SDC transfectants. (**B**) Detected fluorescence intensities of AAV9-untreated SDC transfectants (i.e., SDC ctrls) were normalized to AAV9-untreated WT K562 cells (i.e., K562 ctrl) as standards. The bars represent the mean + SEM of three independent experiments. Statistical significance vs. standards was assessed with ANOVA. Compared to standards, no statistical significance was detected (as assessed with ANOVA).

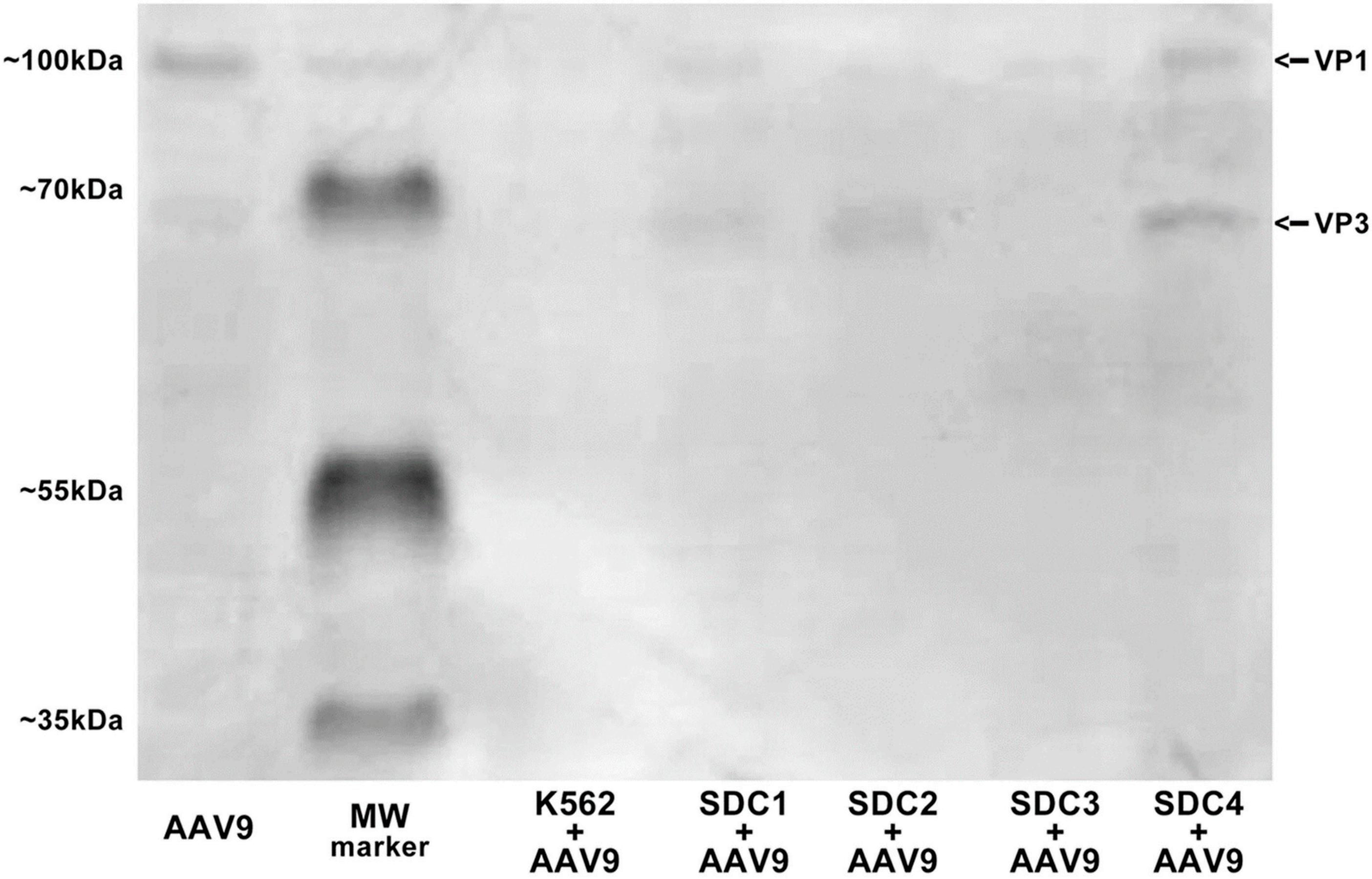

Figure S6. SDS-PAGE showing AAV9 capsid proteins immunoprecipitated with either of the SDC isoforms from extracts of AAV9-treated WT K562 cells and SDC transfectants. Lane 1: 10^6 vg recombinant AAV9; lane 2: MW marker; lanes 3-7: immunoprecipitates of AAV9-GFP-treated WT K562 cells (controls) and SDC transfectants. Standard protein size markers are indicated on the left.

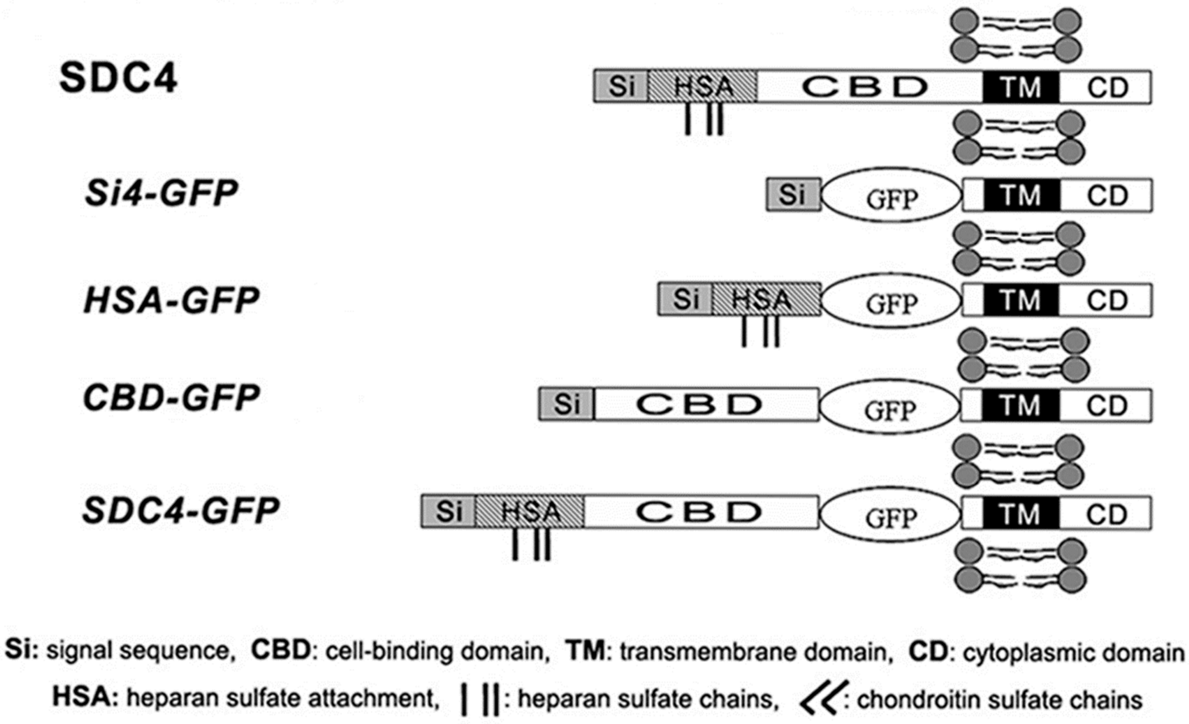

Figure S7. Schematic representation of the SDC4 mutants utilized in the study.

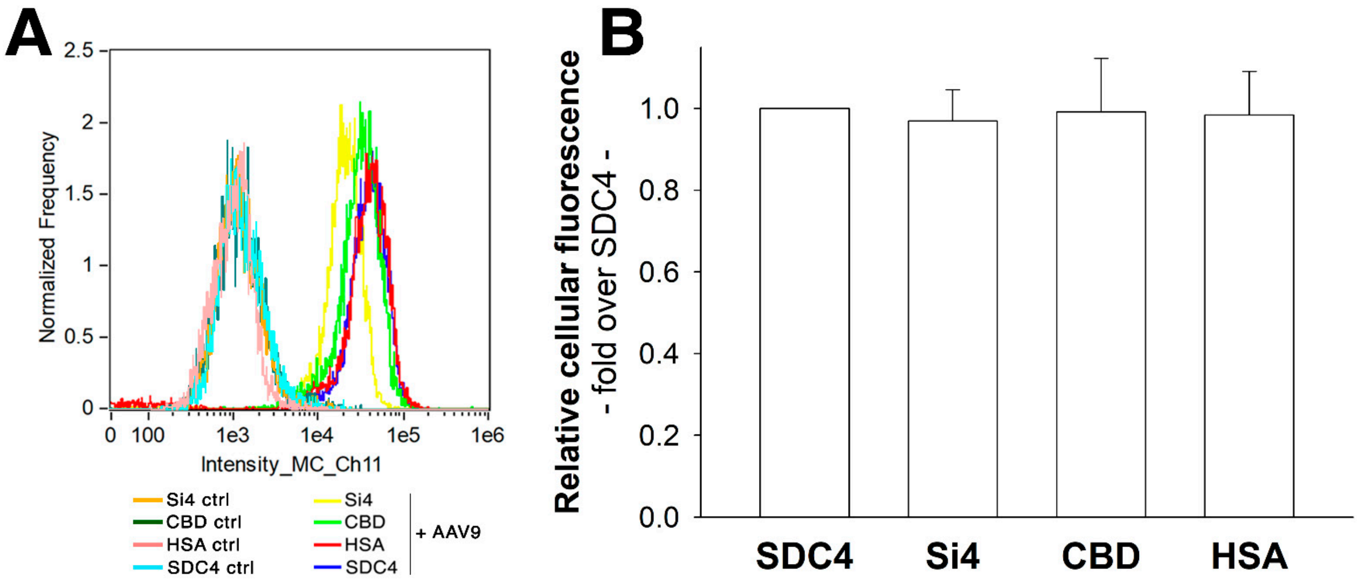

Figure S8. Control studies of AF-633 secondary antibody-treated but AAV9-untreated WT SDC4 transfectants and mutants as measured with flow cytometry. SDC4 mutants incubated with AAV9-GFP vectors at 4 x 10^4 vg/cell for 6h were fixed, permeabilized and treated with specific primary AAV9 and AF 633-labeled secondary antibodies. Control cells untreated with AAV9 were also fixed, and permeabilized but treated with AF 633-labeled secondary anti-mouse antibodies. (**A**) Representative flow cytometry histogram showing the fluorescence of AAV9-treated and untreated SDC4 transfectants and mutants. (**B**) Detected fluorescence intensities of AAV9-untreated SDC4 mutants were normalized to AAV9-untreated WT SDC4 transfectants (i.e., SDC4 ctrl) as standards. The bars represent the mean + SEM of three independent experiments. Statistical significance vs. standards was assessed with ANOVA. Compared to standards, no statistical significance was detected (as assessed with ANOVA).

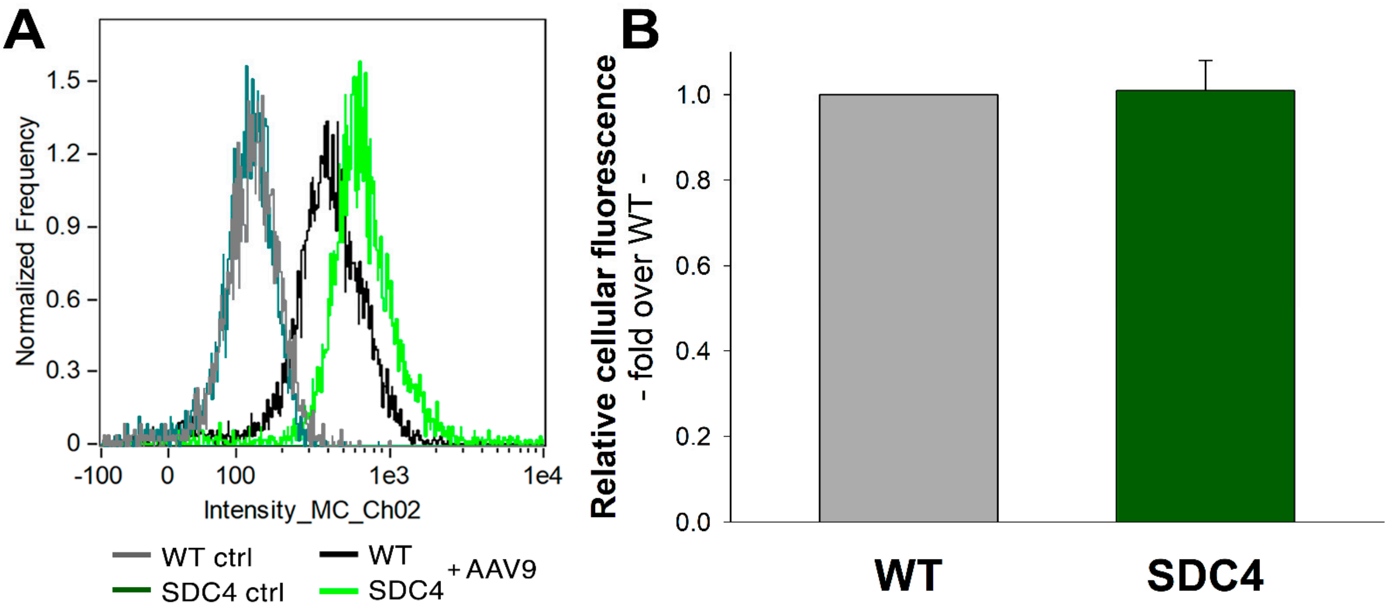

Figure S9. Green autofluorescence of WT SH-SY5Y cells and SDC4 transfectants measured with flow cytometry. (**A**) Representative flow cytometry histogram showing the fluorescence AAV9-treated and untreated (i.e., controls) WT SH-SY5Y cells and SDC4 transfectants. (**B**) Detected fluorescence intensities of AAV9-untreated SDC4 transfectants (i.e., SDC4 ctrl) were normalized to AAV9-untreated SH-SY5Y cells (i.e., WT ctrl) as standards. The bars represent the mean + SEM of three independent experiments. Statistical significance vs. standards was assessed with ANOVA. Compared to standards, no statistical significance was detected (as assessed with ANOVA).

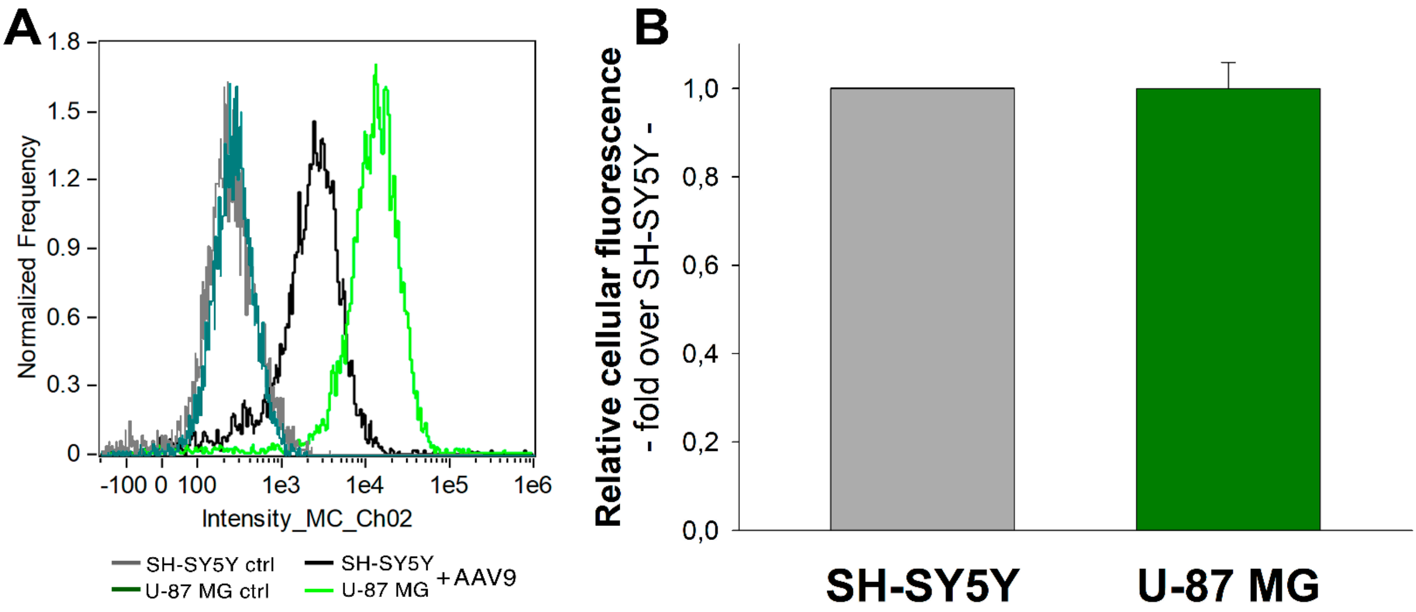

Figure S10. Autofluorescence of WT SH-SY5Y and U-87 MG cells as measured with flow cytometry. (**A**) Representative flow cytometry histogram showing the fluorescence AAV9-treated and untreated (i.e., controls) WT SH-SY5Y and U-87 MG cells. (**B**) Detected fluorescence intensities of AAV9-untreated U-87 MG cells (i.e., U-87 MG ctrl) were normalized to AAV9-untreated WT SH-SY5Y cells (i.e., SH-SY5Y ctrl) as standards. The bars represent the mean + SEM of three independent experiments. Statistical significance vs. standards was assessed with ANOVA. Compared to standards, no statistical significance was detected (as assessed with ANOVA).
